# Cognitive training related changes in fMRI activations: a neural mechanism of transfer to executive functions in normal aging

**DOI:** 10.1002/alz.093461

**Published:** 2025-01-09

**Authors:** Paulina Skolasinska, Shuo Qin, Hyun Kyu Lee, Michelle W Voss, Chandramallika Basak

**Affiliations:** ^1^ University of Texas at Dallas, Richardson, TX USA; ^2^ National University of Singapore, Singapore, Singapore Singapore; ^3^ Posit Science Inc., San Francisco, CA USA; ^4^ University of Iowa, Iowa City, IA USA

## Abstract

**Background:**

Improvement of cognitive functions is often accompanied by changes in brain function after cognitive training in older adults. In this large‐scale dual‐site fMRI study we investigated the effects of BrainHQ cognitive training (CT; ®Posit Science Inc.) and an active control (AC) group on brain activations during a demanding executive control (EC) task among cognitively normal older adults. CT was an adaptive, speed‐based program, with conflict resolution and switching, Participants in the AC arm played non‐timed casual games with low demands on EC. We expected changes in the activations of brain regions related to motor processing and control mechanisms on a hybrid block/event‐related Task‐switching fMRI paradigm alongside improvements in performance.

**Methods:**

We identified brain regions differentially sensitive to the effects of CT and AC interventions using group‐by‐time generalized linear models to determine block‐related (single and dual blocks), and event‐related activations. The latter determined regions sensitive to varieties of cognitive and motor control processes, including local switch cost (switch‐nonswitch trials) and compatibility switch cost (CSC; incompatible‐compatible trials). The resulting activations were subjected to linear mixed models (LMM) to determine post‐training group differences, after controlling for differences in baseline activation.

**Results:**

Compared to AC, CT showed consistently increased activations in the premotor and right prefrontal cortices–regions related to higher‐order motor planning and EC, following the intervention. Increased activation of this region was correlated to gains in Task‐switching accuracy and the EC construct. A CSC‐sensitive region was a large right‐lateralized frontoparietal area including both EC areas and regions involved in sensorimotor function. Post‐training, CT showed lower activation than AC, who showed an increase, but only during the compatible condition. Increased activation observed in AC was maladaptive to cognition as it correlated with reduced gains in the EC construct. Group‐by‐time interactions favoring CT were also observed in cognitive and motor processing cerebellar regions.

**Conclusions:**

Compared to AC, CT showed compensatory adaptive changes in fMRI activations that were beneficial to cognition beyond the fMRI task. Training‐related changes on the Task‐switching paradigm can provide meaningful information on neural underpinnings of transfer to tasks of similar cognitive functions in normal aging.

